# O.4.1-6 Multidimensional physical activity profiles and changes in physical function and self-reported walking ability among community-dwelling older adults – a 4-year follow-up

**DOI:** 10.1093/eurpub/ckad133.171

**Published:** 2023-09-11

**Authors:** Lotta Palmberg, Laura Karavirta, Antti Löppönen, Matti Hyvärinen, Taina Rantanen, Timo Rantalainen

**Affiliations:** Gerontology Research Center and the Faculty of Sport and Health Sciences, University of Jyväskylä, Finland; Gerontology Research Center and the Faculty of Sport and Health Sciences, University of Jyväskylä, Finland; Gerontology Research Center and the Faculty of Sport and Health Sciences, University of Jyväskylä, Finland; Physical Activity, Sports and Health Research Group, Department of Movement Sciences, KU Leuven, Leuven, Belgium; lotta.m.palmberg@jyu.fi; Gerontology Research Center and the Faculty of Sport and Health Sciences, University of Jyväskylä, Finland; Gerontology Research Center and the Faculty of Sport and Health Sciences, University of Jyväskylä, Finland; Gerontology Research Center and the Faculty of Sport and Health Sciences, University of Jyväskylä, Finland

## Abstract

**Purpose:**

Physical activity (PA) is multidimensional but often assessed using single metrics. We studied how data-driven PA profiles predict changes in physical functioning and self-reported walking ability over time among older adults.

**Methods:**

Participants (n = 318) were community-dwelling 75-, 80- and 85-year-olds who wore a thigh-mounted accelerometer for 3-7 days at baseline. PA intensity, fragmentation, absolute and relative PA minutes, sit-to-stand transitions, and gait bout characteristics were used in PA profiling using k-means cluster analysis. Physical function was assessed at baseline and at follow-up 4 years later. Substantial decline in physical function was operationalized as decline of at least 2 points in the Short Physical Performance Battery (SPPB, range 0-12) and at least 0.2 m/s decline in habitual 10-m walking speed. Change in self-reported walking ability during 2 km walk was assessed using a 5-point scale (decline vs. no change/improvement for all). Data were analyzed using logistic regression analysis.

**Results:**

Three physical activity profiles were identified, and named as Exercisers, Actives and Inactives. Exercisers and Actives accumulated high PA minutes, but Actives engaged in predominantly light intensity PA that was accumulated in shorter bouts. The largest difference was observed in relative PA minutes (minutes exceeding participants’ preferred walking speed). Inactives accumulated the lowest PA minutes, intensity and highest activity fragmentation.

During the follow-up period, 32% of participants experienced a substantial decline in walking speed, 24% in SPPB score and 21% in self-reported walking ability. Among women, Exercisers, but not Actives, had lower risk for a substantial decline in walking speed (OR 0.30, 95% CI 0.10-0.95) and SPPB score (0.33, 0.12-0.88) compared to Inactives after adjusting for age, depressive symptoms, comorbidity and baseline walking speed or SPPB score. Among women, Exercisers also had lower risk for decline in self-reported walking ability, but this was explained by differences in health. For men, the differences were not statistically significant.

**Conclusions:**

Daily PA that is accumulated in longer bouts and at intensity exceeding one’s preferred walking speed may be especially beneficial for the maintenance of physical function with advancing age.

**Funding Source:**

Academy of Finland, Finnish Ministry of Education & Culture and European Research Council.

